# Catalyst Protonation Changes the Mechanism of Electrochemical Hydride Transfer to CO_2_

**DOI:** 10.1021/acsorginorgau.4c00041

**Published:** 2024-10-04

**Authors:** Kevin Y. C. Lee, Dmitry E. Polyansky, David C. Grills, James C. Fettinger, Marcos Aceves, Louise A. Berben

**Affiliations:** † Department of Chemistry, University of California, Davis, California 95616, United States; ‡ Chemistry Division, Brookhaven National Laboratory, Upton, New York 11973-5000, United States

**Keywords:** electrocatalysis, carbon dioxide, mechanism, iron, hydride transfer, catalysis, reduction

## Abstract

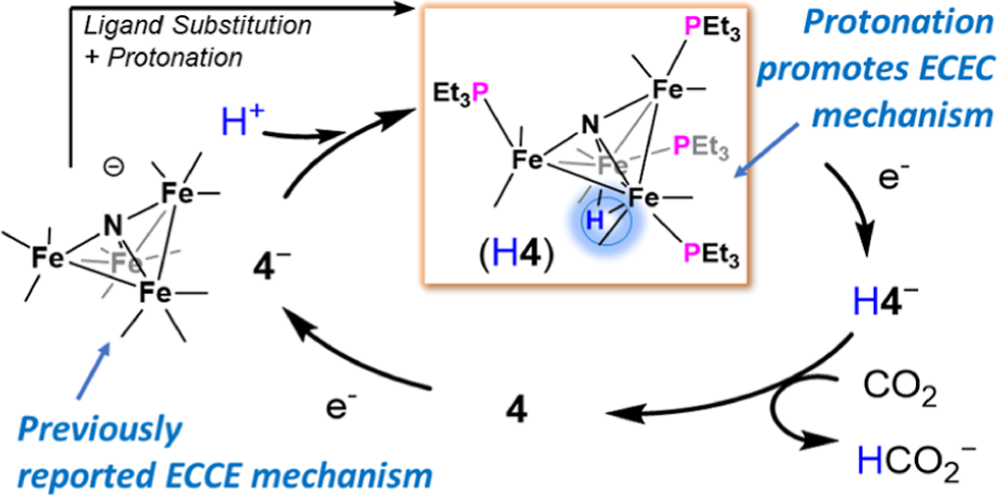

It is well-known that addition of a cationic functional group to a molecule lowers the necessary applied potential for an electron transfer (ET) event. This report studies the effect of a proton (a cation) on the mechanism of electrochemically driven hydride transfer (HT) catalysis. Protonated, air-stable [HFe_4_N(triethyl phosphine (PEt_3_))_4_(CO)_8_] (H**4**) was synthesized by reaction of PEt_3_ with [Fe_4_N(CO)_12_]^−^ (**A**^–^) in tetrahydrofuran, with addition of benzoic acid to the reaction mixture. The reduction potential of H4 is −1.70 V vs SCE which is 350 mV anodic of the reduction potential for **4**^–^. Reactivity studies are consistent with HT to CO_2_ or to H^+^ (carbonic acid), as the chemical event following ET, when the electrocatalysis is performed under 1 atm of CO_2_ or N_2_, respectively. Taken together, the chemical and electrochemical studies of mechanism suggest an ECEC mechanism for the reduction of CO_2_ to formate or H^+^ to H_2_, promoted by H**4**. This stands in contrast to an ET, two chemical steps, followed by an ET (ECCE) mechanism that is promoted by the less electron rich catalyst **A**^–^, since **A**^–^ must be reduced to **A**^2–^ before H**A**^–^ can be accessed.

Improvements to electrochemically driven hydride transfer (HT) reaction performance are needed to target further enhancements in current efficiency, product selectivity and reaction kinetics, pertinent to applications including solar fuels chemistry,^1−8^ and organic electrosynthesis.^9,10^ To access these enhancements to performance it is very helpful to understand the effects of catalyst structure on reaction outcomes and reaction mechanism with molecular level detail. One area where performance improvements are valuable is in the lowering of overpotential required for a reaction, and the reasons for this are twofold: a lowered overpotential enhances the energy efficiency and, a low overpotential is known to enhance the selectivity for a single reaction product. Higher overpotential can promote side reactions leading to decreased efficiency and selectivity in electrochemical processes.^11^

Addition of a cation to the primary coordination sphere or secondary coordination sphere (SCS) of an electrocatalyst lowers the necessary applied potential for an electron transfer (ET) event.^12,13^ Strategies by others also demonstrate that intelligent placement of the cationic functional group can promote specific interactions with the substrate to enhance rate while lowering overpotential.^14^ Notably, placement and number of cations in the SCS is important, and more cations does not always result in more efficient catalysis.^14,15^ Most additions of cationic functional groups to the SCS are achieved through installation of alkyl-ammonium,^14−16^ or imidazolium groups,^17^ or alkali and alkaline earth cations encapsulated by crowns ethers.^18−20^

In this report we discuss protonation of a HT electrocatalyst as a simple strategy to add a positive charge to the electrocatalyst and thereby lower the applied potential for an ET event by ∼400 mV. We have previously reported on the effect that a proton has on ET only for Et_4_N[Fe_4_N(CO)_12_] (Et_4_N-**A**, Chart 1), and shown that reduction is shifted anodically by 320 mV without changing the electronic properties of the central metal catalyst core, as measured by the energy of the CO absorption bands in the infrared (IR) spectra.^21^ In the presence of a proton donor, **A**^–^ can reduce CO_2_ to formate (HCO_2_^–^) at 95% Faradaic efficiency (FE) when a potential of −1.2 V vs SCE is applied.^22^

**Chart 1:**
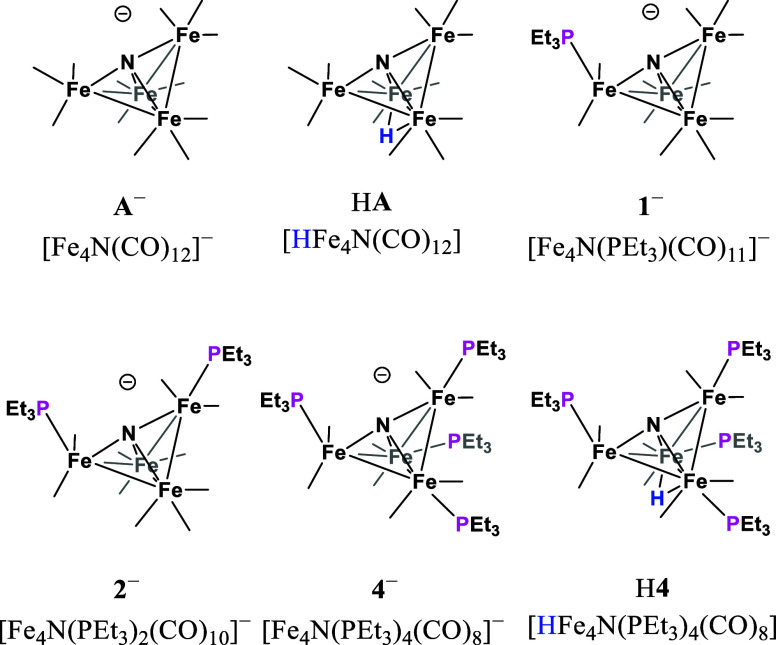
Compound numbering system used in the text. The cation in each case is Et_4_N^+^. Compounds **A**^–^, H**A**, **1**^–^, **2**^–^, and **4**^–^ have been previously reported.^13,22^

The new contribution in this work is to look at the effects of a proton on the catalyst reactivity. Since H**A** has a low p*K*_a_ and is not stable to deprotonation in polar solvents, we chose a more electron rich derivative of **A**^–^ so that the protonated cluster does not dissociate in MeCN solution. From the series of triethylphopshine (PEt_3_)-substituted clusters including [Fe_4_N(CO)_11_(PEt_3_)]^−^ (**1**^–^), [Fe_4_N(CO)_10_(PEt_3_)_2_]^−^ (**2**^–^), and [Fe_4_N(CO)_8_(PEt_3_)_4_]^−^ (**4**^–^), we targeted H**4** since it is the most electron rich (Chart 1). For H**4**, we determine that the mechanism for formate or H_2_ evolution under 1 atm of CO_2_ or N_2_, respectively, proceeds via initial ET to give (H**4**)^−^. This is followed by a chemical step that is HT to a proton or CO_2_ to afford a mixture of H_2_ and formate (Scheme 1). Overall, the mechanism for formate or H_2_ formation by H**4** follows an ECEC pathway, where E = electrochemical step, and C = chemical step. The mechanism differs from that reported for **A**^–^, where ET and proton transfer (PT) steps are needed before formate formation, and the overall pathway is ECCE (or EECC, which is equivalent).

**Scheme 1:**
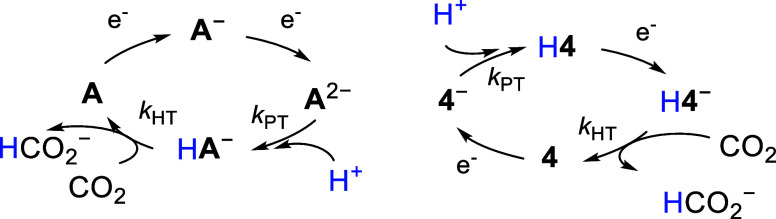
Proposed Mechanisms for Formate (HCO_2_^–^) Formation under 1 atm CO_2_ (Left) by **A**^–^, **1**^–^, and **2**^–^ Following an ECCE Mechanism; and (Right) by H**4** Following an ECEC Mechanism1 The catalytic cycle for both schemes starts at the top.

## Results and Discussion

**Scheme 2:**
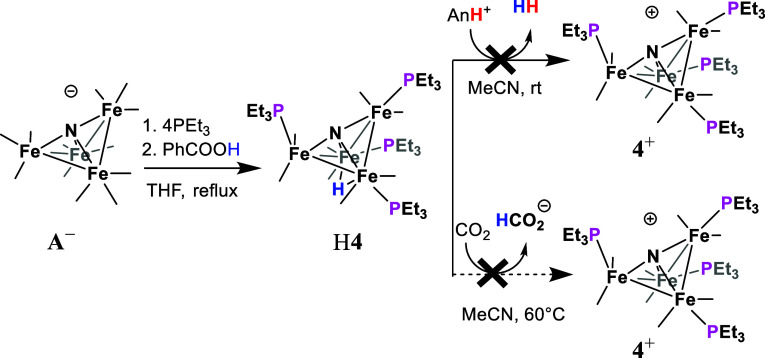
Synthesis of H**4** and Reaction Chemistry with Brønsted Acids and CO_2_

We have previously observed H**4** as an intermediate in the synthesis of **4**^–^ and so we set out to isolate H**4** for this study.^13^ Tetra substituted H**4** was synthesized by reaction of **A**^–^ with 6 equiv of PEt_3_ at reflux in tetrahydrofuran (THF) with 1.2 equiv of benzoic acid over 24 h (Scheme 2). After removal of THF in vacuo, the resulting solid was washed with water to remove the benzoate and recrystallized from a saturated solution of hexane to afford H**4** in 80% yield as relatively air-stable, dark block-shaped crystals. Characterization of H**4** was performed using IR, ^1^H, ^13^C, and ^31^P NMR spectroscopy along with combustion analysis (Figures S1–S5). The IR spectrum of H**4**, collected in THF solutions, showed three υ_CO_ absorption bands at 1940, 1930, and 1900 cm^–1^, which are at lower energies than those of **A**^–^. This shift is consistent with a more electron rich cluster core after replacement of four CO ligands with four PEt_3_ ligands.^23,24^ There are 3 bands for H**4** as compared with two bands for **A**^–^, consistent with the lower symmetry of H**4** (Figure S1). The proton NMR spectrum of H**4** collected in CDCl_3_ shows a distinctive triplet of triplets at −28.3 ppm, which arises from coupling of the proton on the surface of the cluster with the two pairs of unique phosphorus atoms in the molecule (Figures S3 and S4). The hydride on previously reported H**A** appears at −31.2 ppm, which is 3 ppm downfield of H**4**.^25^ Two resonances are observed in the ^31^P NMR spectrum at 42.2 and 42.8 ppm and these are shifted downfield by 61.7 and 62.3 ppm, respectively, relative to PEt_3_ which appears at −19.5 ppm. Resonances for **4**^–^ are observed at 42.8 and 42.2 ppm, which is within 0.2 ppm of H**4**, suggesting that the H atom on the cluster does not significantly affect the core electronic properties relative to the unprotonated cluster. The ^13^C NMR spectrum shows two doublets at 22.5 and 20.5 ppm, and a doublet at 8.2 ppm and these are assigned to the CH_2_ and CH_3_ on the PEt_3_ ligands. Singlet resonances at 225.3, 223.6, 220.9, and 216.1 ppm are attributed to carbonyl ligands (Figure S5).

**Figure 1:**
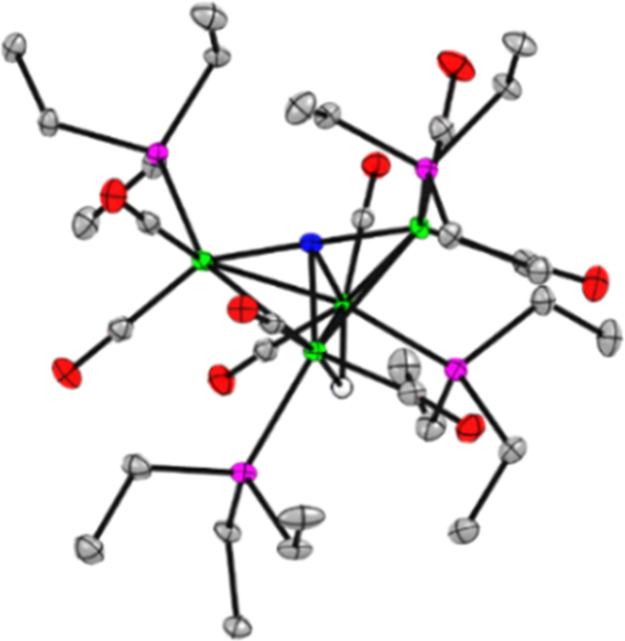
Solid-state structure of H**4**. Green, blue, pink, gray, and red ellipsoids, and white sphere represent Fe, N, P, C, O, and H atoms, respectively. Ellipsoids shown at 50%; H atoms omitted except for hydride.

Dark block-shaped crystals grown from a saturated hexane solution over 3 days were used to determine the solid-state structure of H**4** using single crystal X-ray diffraction (Tables S1 and S2, Figures 1 and S6). The solid-state structure of H**4** shows a H atom bridging the hinge of the butterfly shaped cluster, and the H atom was located in the difference map. It has been previously reported that protonation of singly anionic **A**^–^ also occurs at the hinge regardless of other ligand substitutions patterns of the CO ligands.^26,27^ The Fe–Fe hinge bond length appears to be shortest with the strongest π-accepting ligands: in H**4** it is 2.601(5) Å, compared with 2.521(1) Å in (H**A**)^−^,^22^ or 2.5771(6) Å in [HFe_4_N(CO)_8_(CNAr^Mes2^)_4_], where Ar is aryl and Mes is mesityl.^27^ The PEt_3_ ligands in H**4** are evenly distributed about the core, with one ligand on each Fe atom, and this pattern is likely driven by the steric effects of the moderate cone angle of PEt_3_ (132°). Bulkier substituents such as MePTA^+^ and CNAr^Mes2^, are known to form unevenly tetra-substituted clusters where two ligands fit at the wingtip Fe atoms so that one hinge Fe is not substituted.^13,27^ Although attempts to obtain a crystal structure for **4**^–^ have been unsuccessful, we can elucidate the positions of the PEt_3_ ligands via ^31^P NMR spectroscopy. The spectrum of **4**^–^ has two unique resonances, which is consistent with one PEt_3_ ligand on each Fe center.^13^

### Reaction Chemistry of H**4**

**Figure 2:**
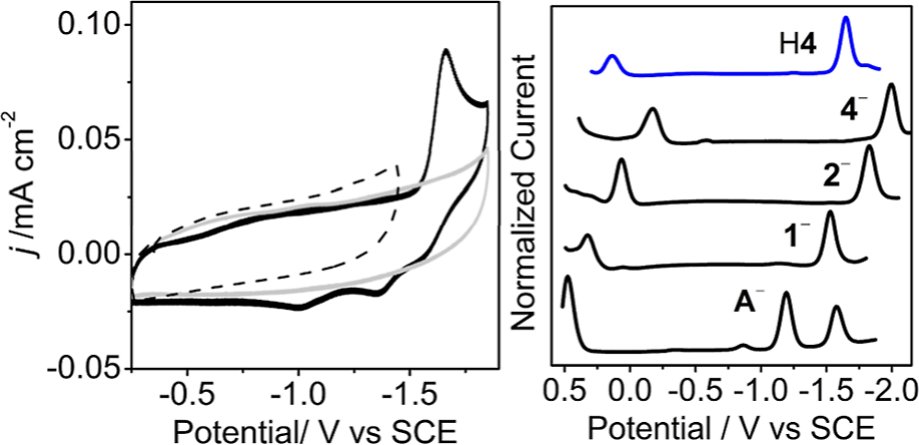
(Left) CV’s of 0.1 M Bu_4_NBF_4_ MeCN solution under 1 atm N_2_ at 0.1 V s^–1^ (gray); with added 0.1 mM H**4** (black); and with the scan direction reversed at −1.5 V (dotted). (Right) Normalized DPV’s of H**4** (blue) and of **4**^–^, **2**^–^, **1**^–^, and **A**^–^ Glassy carbon (GC) working electrode.

As a first step toward understanding the mechanisms for reaction of H**4**, we probed a series of chemical reactions designed to assess both the acidity and the hydricity of H**4** (Scheme 2). IR spectra of H**4** dissolved in either MeCN or toluene appear unchanged and so we know that H**4** remains intact in solution. No reaction between H**4** and sodium phenolate (p*K*_a_(PhOH) = 29.2 in MeCN),^28^ was observed, and with KO^*t*^Bu (p*K*_a_(^*t*^BuOH) = 45.2 in MeCN),^29^ we observed formation of **4**^–^ using IR spectroscopy. Based on the available experimental data, we bracket the p*K*_a_ of H**4** to be 29 < p*K*_a_ < 45. For comparison, we determined the p*K*_a_ for H**A** based on the equilibrium between **A**^–^ and H**A**, which is obtained when H**A** is dissolved in MeCN solution. For H**A**, we quantified the solution equilibria using IR absorption spectroscopy and Beer’s Law, which showed that the p*K*_a_ for H**A** is 3.6 (Figure S7). This is a significantly lower p*K*_a_ value for H**A**, compared to the p*K*_a_ of H**4**, as expected since the cluster core in H**4** is far more electron rich. When H**4** is dissolved in MeCN it does not dissociate (Figure 2 right).

Hydricity (Δ*G*_H–_) is the free energy change for loss of hydride, and knowledge of this value can predict whether a hydride reacts with some substrates. The hydricity of H**4** was probed by studying its reactions with several acids, including benzoic acid (PhCOOH, p*K*_a_ = 20.3 in MeCN), and anilinium (AnH^+^, p*K*_a_ = 10.6 in MeCN),^28^ while the reaction was monitored for H_2_ evolution by analysis of the reaction headspace with gas chromatography and a thermal conductivity detector (GC-TCD). No evolution of H_2_ was observed with either acid when one equivalent of the acid was added. No reaction of H**4** with AnH^+^ implies that Δ*G*_H–_(H**4**) > 62 kcal mol^–1^ (Calculation S1),^30^ and that reduction of H**4** to (H**4**)^−^ will be needed to enter into a productive catalytic cycle that includes HT to CO_2_. Indeed, a reaction of H**4** with CO_2_ did not yield formate over 24 h of heating at 60 °C in MeCN-*d*_3_, nor in MeCN/H_2_O (95:5), as monitored using proton NMR spectroscopy. The hydricity of formate is known to be 44 kcal mol^–1^ in MeCN.^31^

### Electrochemical Measurements under 1 atm N_2_

Cyclic voltammetry (CV) and differential pulse voltammetry (DPV) measurements were performed on 0.1 M Bu_4_NBF_4_ MeCN solutions of 0.1 mM H**4** (Figure 2, Table 1). CVs collected under 1 atm N_2_ showed a reduction event (*E*_pc_) at −1.70 V vs SCE and −1.65 V vs SCE for DPV. On the return oxidative scan of the CV experiments, two oxidation events with *E*_pa_ of −1.40 and −1.10 V were observed. Relative to the half-wave potential (*E*_1/2_) for [HFe_4_N(CO)_12_], reported at −0.57 V,^22^ the reduction of H**4** is shifted cathodically by −1130 mV by the four electron donating PEt_3_ groups which have replaced four CO ligands. A series of comparisons which illustrates the effects of PEt_3_ and H^+^ substitution on the clusters can be obtained from comparison with our previously reported data on the characterization of **1**^–^, **2**^–^ and **4**^–^, which have *E*_pc_ of −1.53, −1.83, and −2.0 V, respectively, measured using CV or DPV (Figure 2 right).^13^ Each of the first two PEt_3_ additions result in a 300 mV cathodic shift per added PEt_3_ ligand relative to **A**^–^. Addition of two more PEt_3_ ligands in **4**^–^ provides a further 250 mV cathodic shift. For H**4**, *E*_pc_ is at −1.65 V and this is 350 mV anodic of the unprotonated analog, **4**^–^, and 100 mV anodic of the twice-substituted **2**^–^. Protonation is therefore a useful tool for accessing milder reduction potentials.

**Table 1:** *E*_pc_ Values from CV and DPV^a^

	*E*_pc_/V, CV	*E*_pc_/V, DPV	*E*_1/2_/V, PR
**A**^–/2–^	–1.23^b^	–1.23	–1.24
**1**^–/2–^	–1.55^b^	–1.53	nd
**2**^–/2–^	–1.87^b^	–1.83	nd
**4**^–/2–^	–2.05^b^	–2.0	nd
(H**4**)^0/–^	–1.70	–1.65	–1.71^c^
			–1.68^d^

a PR Determination of *E*_1/2_. All Potentials are vs SCE.

b Species present 1 μs after an electron pulse.

c From ref (13). nd = not determined. DPV = differential pulse voltammetry, PR = pulse radiolysis.

d Species present 1 μs after an electron pulse.

To probe the origin of the oxidation events in the CV for H**4** at −1.4 V and at −1.10 V, we performed a reductive scan that turned around at −1.5 V and this showed no oxidative redox events on the return scan, suggesting that the events with *E*_pa_ of −1.40 and −1.10 V are associated with oxidation of the species generated by reduction at −1.70 V (Figure 2). It is well-documented that the reduction of metal carbonyl clusters can afford multiple species observed in the oxidative return trace due to fluxionality of the capping ligands that move around the surface of the cluster without decomposition of the cluster.^32^ Our previous reports of related phosphine-substituted derivatives of **A**^–^, including [Fe_4_N(CO)_11_(PPh_3_)]^−^ and [Fe_4_N(CO)_11_(PPh_2_EtOH)]^−^,^23^ also show that the return oxidative event shifted anodically from the expected potential predicted by a simple reversible redox couple. In those prior examples, IR spectroelectrochemical (IR-SEC) experiments showed full regeneration of the cluster upon redox cycling, which confirms there is no decomposition.^33^ Here, we could not perform IR-SEC experiments because the more negative *E*_p_ value for H**4** leads to background H_2_ evolution from the weak acid H**4** at the Au electrode of the IR-SEC cell.

As an alternative approach for the determination of the *E*_1/2_ of H**4**, we performed an experiment that uses pulse radiolysis coupled with time-resolved infrared spectroscopy (PR-TRIR).^34^ Pulse radiolysis utilizes high-energy electron pulses from an accelerator to excite a sample, and it permits the rapid one-electron reduction (or oxidation) of a dissolved solute. In this case, a mixture of the sample under investigation and a standard was reduced, so that their equilibrium redox composition, and therefore the *E*_1/2_ for the sample could be determined by comparison with the *E*_1/2_ of the standard (see Experimental Section for more details, and Calculation S2). As an initial control experiment, the *E*_1/2_ for **A**^–^ was determined to be −1.238 ± 0.004 V using a TRIR measurement recorded 20 μs after the electron pulse (Figure S8, Table 1): this agrees with the value of −1.23 V measured using DPV.

The PR-induced reduction of H**4** results in the disappearance of three υ_CO_ bands at 1943, 1934 and 1904 cm^–1^, displayed as negative peaks in Figure S9C. Concomitantly with the disappearance of H**4**, a new set of peaks is observed around 1922, 1913 and 1880 cm^–1^, 1 μs after the electron pulse. This is consistent with the shift of υ_CO_ vibrations to lower energy due to increased electron density on the cluster leading to increased π back-donation. The reduced cluster evolved into a new species after 20 μs, exhibiting a new set of carbonyl vibrations around 1920, 1910 and 1895 cm^–1^, which are consistent with a different form of the reduced cluster (Figure S9C). PR-TRIR equilibrium experiments performed with H**4** provided an *E*_1/2_ = −1.707 ± 0.003 V for the species detected 1 μs after the electron pulse, and −1.681 ± 0.002 V for the second species that is formed after 20 μs (Figure S9, Table 1). We do not resolve both of these reduction events in the DPV experiment, but the DPV measurement of −1.70 V for *E*_1/2_ is in good agreement with the average of the two redox couples obtained from PR-TRIR (Figure 2, Table 1).

The observation of two reduced species by PR-TRIR is consistent with the high fluxionality of the CO and PEt_3_ ligands on the surface of the cluster, which leads to more than one isomer in solution. The fluxional behavior of CO and phosphine ligands in multimetal complexes is well-established.^35−37^ CV’s collected under 1 atm CO did not show an increase in reversibility, and this supports the assignment of the new species to a fluxional process rather than dissociation of CO from the cluster (Figure S10). An analogous experiment where up to 23.4 mM PEt_3_ was added to a solution of H**4** resulted in no change in the CV, supporting our assessment for fluxional CO ligands (Figure S10).

### Electrochemical Measurements under 1 atm CO_2_

**Figure 3:**
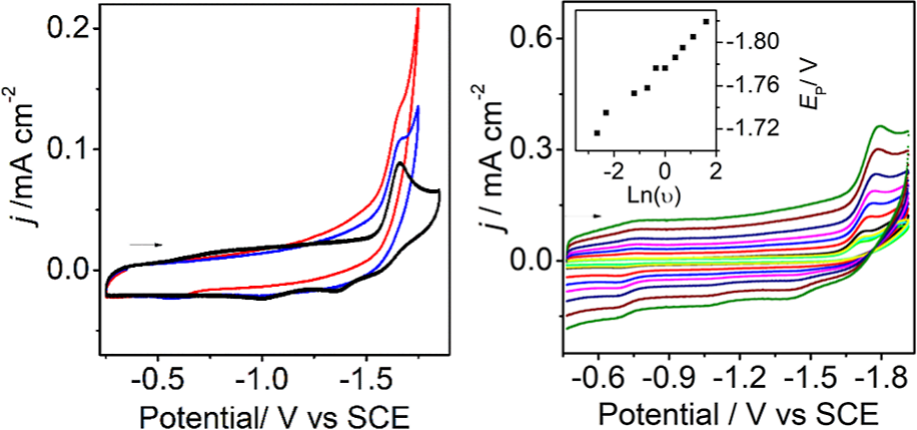
CV’s of 0.1 mM H**4** in 0.1 M Bu_4_NBF_4_ MeCN: (left) under N_2_ (black), in MeCN/H_2_O (95:5) under N_2_ (blue), and under 1 atm CO_2_ in MeCN/H_2_O (95:5) (red) at 0.1 V s^–1^; (right) variable scan rate data collected under 1 atm CO_2_ from 0.1–1 V s^–1^ suggests HT following ET. Insets: Plot of *E*_P_ vs scan rate (υ). GC working electrode.

We next performed CV experiments with added water to start understanding the interactions of reduced H**4** with protons. Addition of 2.78 M (5%) water to the CV experiment resulted in an anodic shift of 40 mV relative to H**4**, a small current increase at the reduction event (*i*_c_/*i*_p_ = 2), and the loss of return waves following reduction at −1.70 V (Figure 3 left). A CV of the same solution collected under 1 atm CO_2_ showed a further slight current increase. This pattern is consistent with H_2_ production under N_2_, and formate or H_2_ production under CO_2_.^22^

Controlled potential electrolysis (CPE) experiments were performed with H**4** in MeCN/H_2_O (95:5) under 1 atm CO_2_ to characterize the products formed (Calculations S2, S3, Table S3, Figures S11–S13). From CPE experiments run at −1.52 V, an analysis of the headspace using GC-TCD revealed H_2_ evolution with 50% FE, and formate was detected with 46% FE using ^1^H NMR spectroscopy. CPEs performed on rinsed electrodes in a solution of fresh MeCN/H_2_O (95:5) showed no formate formation. The coproduction of H_2_, along with the formate formation, is consistent with background proton reduction at the GC electrode, since the CPE is run at −1.52 V. The blank experiment run under 1 atm CO_2_ in the absence of added H**4** produced H_2_ although only half the amount of charge was passed. Under 1 atm N_2_, an equivalent set of CPE’s run at −1.52 V showed that H_2_ is formed in quantitative FE (Table S4 and Figure S12). No formate is observed under 1 atm N_2_. To further confirm that the formate product is derived from CO_2_, we performed a CPE experiment using 1 atm of ^13^CO_2_ and confirmed the production of formate using ^13^C NMR spectroscopy (Figure S14). We also checked for decomposition of H**4** during CPE by looking for CO using headspace analysis via GC-TCD, and no CO was observed down to a detection limit of 1000 ppm of CO. Solution analysis performed after the CPE experiment using IR spectroscopy showed that the CO region of the IR spectrum remains unchanged and this is also consistent with the stability of H**4** (Figure S15).

### Studies of Formate Formation Mechanism

We next considered the mechanism of formate or H_2_ formation by H**4**. As a starting point, we know from the stoichiometric chemical reactions described earlier that H**4** does not react directly with CO_2_ in MeCN/H_2_O (95:5) even when heated up to 60 °C (Scheme 2). The initial step in the proposed mechanism for formate formation is likely therefore ET to afford (H**4**)^−^ followed by HT to CO_2_ or H^+^ which produces formate/H_2_ and **4**, before ET and PT steps regenerate H**4** (Scheme 1). As a test of the proposal that a chemical step follows formation of (H**4**)^−^, a series of CV scans over the range 0.1–1 V/s, under 1 atm CO_2_ were performed. An anodic shift in E_p_ was observed, and this is consistent with a chemical reaction following the ET (Figure 3 right). A possible chemical reaction is HT from (H**4**)^−^ to CO_2_ (eq 1), but alternative pathways or background reactions are dimerization of (H**4**)^−^ to form H_2_ and **4**^–^, reaction of (H**4**)^−^ with H^+^ to form H_2_, or reaction of (H**4**)^−^ with H**4** to form H_2_ (eqs 2–4):

Proposed mechanism
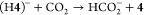
1

Alternate mechanisms
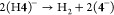
2
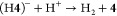
3
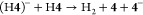
4Under 1 atm N_2_, a cathodic shift with increasing scan rate is also observed and this is consistent with the reactions shown in eqs 2–4 (Figure S16). Taken together, the observations on the reactivity of H**4**, under 1 atm N_2_ or CO_2_ to afford H_2_ or formate, respectively, are consistent with a mechanism involving four elementary steps in the order ECEC. In contrast, **A**^–^ is known to promote an ECCE mechanism for H_2_ or formate formation.^22,45^

The orders of the reaction with respect to [H^+^] and to [catalyst] were studied under atm N_2_ and CO_2_, as further probes of the proposed mechanism for H_2_ and formate formation, respectively. CVs were recorded with successive additions of H_2_O over the range 0.05–2.7 M and the linear correlation of *j* with [H_2_O] suggests a second order reaction in [H^+^] (Figure S17). In a similar experiment, a first order dependence on [H**4**] was established (Figure S18). The order with respect to [CO_2_] could not be definitively determined due to the high background current from H_2_ evolution observed in the CV.

Each catalyst has a rate constant for the observed rate (*k*_obs_/s^–1^) of product formation, which is dependent on a variety of factors like experimental conditions and mechanism.^38^ To obtain a maximum value for *k*_obs_, CVs are generally recorded under reaction conditions where the substrate is not depleted during the CV scan so that a steady-state current is reached. In the present report for H**4**, *E*_p_ is −1.70 V and, as observed in the CPE experiments, this cathodic potential results in significant background H_2_ evolution and a resulting low formate formation FE of 50%. An accurate measurement of the formate formation rate, *k*_obs_ is not possible using a limiting current analysis since the observed current arises from background H_2_ evolution, formate formation, and capacitive current; and these cannot be accurately deconvoluted.

A rough estimate for the hydricity of H**4**^–^ can be made knowing how the hydricity of hydrides of similar structure correlate with their reduction potential,^39^ and charge states of a metal hydride have a dramatic effect on the corresponding hydride.^40^ Hydricity values have been measured for the corresponding hydrides of **A**^–^ and [Fe_4_N(CO)_11_(PPh_3_)]^−^, which are 49,^22^ and 44 kcal/mol,^23^ respectively. Based on the linear trend between reduction potential (*E*_p_) and hydricity,^41^ we estimate H**4**^–^ to have a hydricity of approximately 42 kcal/mol.

### Mechanistic Context

**Figure 4:**
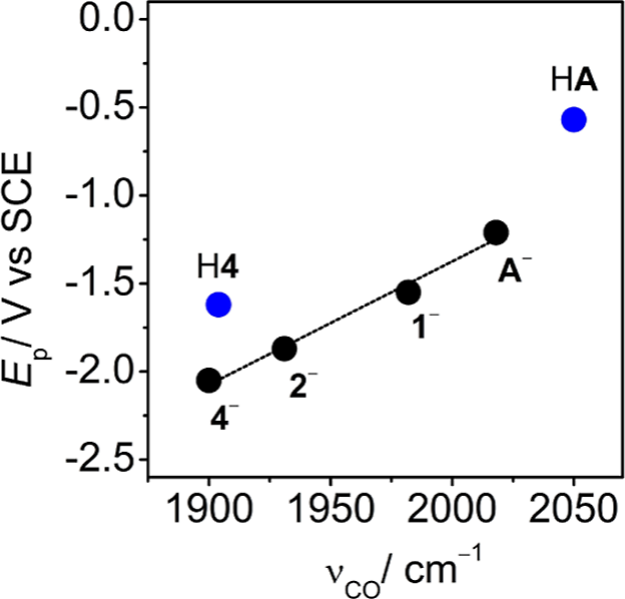
Correlation between *E*_p_ vs ν_CO_; for **A**^–^, **1**^–^, **2**^–^, **4**^–^ (black), and H**4**, and HA (blue). *E*_p_ values obtained from DPV experiments (Figure 2 right and ref (13)).

To choose comparison and discussion points for the reactivity and electronic properties of H**4**, we studied the plot of *E*_p_ vs ν_CO_ for **A**^–^, **1**^–^, and **2**^–^, since *E*_p_ from DPV provides a comparison of catalyst operating potential while ν_CO_ provides a measure of the cluster core electronic properties. We have previously reported that this plot is a straight line for unsubstituted clusters, but addition of cationic phosphine ligands as SCS functional groups leads to deviations from the line.^21^ For H**4**, this same relationship is observed, where H**4** and **4**^–^ have ν_CO_ within 10 cm^–1^, at 1942 and 1951 cm^–1^, respectively. This is compared with a more significant change in *E*_p_ where H4 is reduced more anodically than **4**^–^ by 400 mV. Clusters H**4** and **1**^–^ have the most similar *E*_p_ from DPV values of the series shown, at −1.65 and −1.53 V, respectively (Figure 4). For comparison, the FE for H_2_ and formate evolution under 1 atm CO_2_ by **1**^–^ and **2**^–^ were determined (Table S3), and as in the case of H**4**, the selectivity for formate formation by **1**^–^ and **2**^–^ is low at 50 and 28%, respectively, which is attributed to increased HER at the GC electrode in the same way that poor selectivity for formate formation by H**4** was reported above. For **A**^–^, the selectivity of formate formation is much higher and close to quantitative, which is because the *E*_pc_(**A**^–/2–^) is more anodic where the GC electrode does not interfere (Table S3).

Under HER conditions with a HT mechanism, the rds is often hydride formation,^42^ and this is true for both H**4** and **1**^–^ in the HER reaction.^43^ For formate formation, it is usually HT to CO_2_ which is the rds and we have demonstrated this for **A**^–^ and for **1**^–^ in prior work.^43^

## Conclusion

In this report we have explored the effect of catalyst protonation on the mechanism of electrocatalytic formate formation. The reduction potential for protonated H**4** is 400 mV anodic of **4**^–^. Mechanistic studies performed using CV are consistent with a proposed mechanism where ET initially produces (H**4**)^−^, which then reacts with CO_2_ to give formate and **4**. Further ET and PT steps regenerate H**4**. This is an ECEC mechanism, which is different from previously reported ECCE mechanisms that are known for the [Fe_4_N(CO)_12_]^−^ family of electrocatalysts, and where the rds is HT to CO_2_.

## Experimental Section

### Preparation of Compounds

All manipulations were carried out using standard Schlenk or glovebox techniques under a dinitrogen atmosphere. Unless otherwise noted, solvents were deoxygenated and dried by thorough sparging with Ar gas followed by passage through an activated alumina column (Pure Process Technology). Deuterated solvents were purchased from Cambridge Isotopes Laboratories, Inc., and were degassed before use. All reagents were purchased from commercial vendors and used without further purification. Compounds [Na(diglyme)_2_][Fe_4_N(CO)_12_] (Na(diglyme)_2_-**A**),^44^ Et_4_N[Fe_4_N(CO)_12_] (Et_4_N-**A**),^45^ Et_4_N[Fe_4_N(CO)_11_(PEt_3_)] (Et_4_N-**1**), Et_4_N[Fe_4_N(CO)_10_(PEt_3_)_2_] (Et_4_N-**2**) were synthesized by previously published methods.^13^

### [HFe_4_N(CO)_8_(PEt_3_)_4_] (H**4**)

A 50 mL oven-dried Schlenk flask was charged with 500 mg (0.578 mmol) Na(diglyme)_2_-**A**, 20 mL THF, and 511 μL (3.47 mmol) PEt_3_, in that order. The resulting solution was heated at reflux for 2 h, using an oil bath at reflux for 2 h. After 2 h, 84.72 mg (0.693 mmol) benzoic acid was added to the reaction mixture under dinitrogen. Then the THF solution was heated at reflux overnight, before the solvent was removed under vacuum. The resulting deep brown precipitate was washed twice with 10 mL of water to remove all the salts, and then the product was dissolved in hexane and filtered through Celite. A concentrated hexane solution was kept at −16 °C for 3 days to afford analytically pure brown crystals suitable for X-ray diffraction. Yield: 192 mg (47%). ^1^H NMR (400 MHz, C_6_D_6_): 2.10–1.85 (m, 24H), 1.14 (dt, *J* = 15.1, 7.7 Hz, 18H) 1.05 (dt, *J* = 14.9, 7.5 Hz, 18H), −28.10 (tt, *J* = 27.1, 3.9 Hz, 1H); ^31^P NMR (400 MHz, C_6_D_6_): 42.82, 42.19; ^13^C{^1^H} NMR (400 MHz, C_6_D_6_): δ 22.38 (d, *J*_PC_ = 23.9 Hz) 20.5 (d, *J*_PC_ = 21.6 Hz, PCH_2_CH_3_), 8.70–7.51 (dd, PCH_2_CH_3_). IR (MeCN): ν_CO_ 1942(s), 1933 (s), 1904(m). Anal. Calcd C 41.10, H 6.58, N 1.50; Found: C, 41.22, H 6.52, N, 1.44.

### Electrochemical Measurements

Cyclic voltammograms were recorded under a dinitrogen (Praxair, 99.998%) or carbon dioxide (obtained from crushed dry ice) atmosphere using a CH Instruments Electrochemical Analyzer model 1100C or 620D, a glassy carbon button working electrode (CH Instruments, nominal surface area of 0.0707 cm^2^), a platinum wire auxiliary electrode and an Ag/AgNO_3_ reference electrode with a Vycor tip. Reported potentials are all referenced to the SCE couple and were determined using ferrocene as an external standard where *E*_1/2_ ferrocene/ferrocenium is 0.400 V vs SCE in acetonitrile. For PT measurements, the stability of the reference electrode was confirmed with an internal ferrocene reference at the conclusion of each experiment. Bu_4_NBF_4_ was prepared according to the literature and recrystallized two times using ethyl acetate/hexane.^46^ Unless otherwise noted, all CV and CPE experiments were performed at room temperature, 25 °C. CV’s were plotted using the polarographic convention. Arrow in CV’s indicate starting point and direction of scan.

### Pulse Radiolysis with Time-Resolved IR Detection

PR-TRIR experiments were conducted using a custom-built flow mixing system. All the parts of the flow system were thoroughly dried in a vacuum oven and assembled inside a N_2_ glovebox. All samples were prepared inside a glovebox using dry acetonitrile and the solutions were loaded into gastight syringes. The fully assembled flow setup was transferred from the glovebox to the beamline of the LEAF electron accelerator.^47^ Tunable, continuous wave external-cavity quantum cascade lasers were used as the IR probe light (DRS Daylight Solutions, models MIRcat-QT-2400 and 21052-MHF). The irradiated solution was replaced with freshly mixed solution by flowing a new 0.2 mL aliquot through the cell (2 mm path length). Only one kinetic trace per cell fill was measured. Detailed descriptions of the flow system and PR-TRIR experiments can be found elsewhere.^48,49^

### CPE

(CPE) experiments were performed in a custom designed gastight glass cell under 1 atm of CO_2_ (obtained from crushed dry ice) or 1 atm N_2_. Electrolyte solutions of 0.1 M Bu_4_NBF_4_ MeCN/H_2_O (95:5) were sparged with the respective gas for 30 min prior to the commencement of the experiment. The counter electrode compartment was separated from the working electrode compartment by a glass frit of medium porosity. In a typical experiment, 20 mL of electrolyte solution were used in the working electrode compartment and 25 mL of electrolyte were used in the counter electrode compartment. The working electrode was a glassy carbon plate (Tokai Carbon) with a nominal surface area when immersed in solution of 8 cm^2^. The auxiliary electrode was a coiled Pt mesh (BASi). A stir plate set to 850 rpm was used to stir a 1 cm stir bar in the cathode compartment. Gas measurements were performed using a gastight syringe (Vici) to inject 0.100 mL gas samples into a Varian 3800 gas chromatograph equipped with a thermal conductivity detector. Gas samples were extracted from a sparged, septum-capped side arm on the working electrode compartment. No carbon monoxide, methane, ethane, or ethylene were detected. Before CPE experiments, the cell and electrodes were cleaned and sonicated with 5% nitric acid (aq) for 15 min, rinsed, cleaned twice with distilled water, and oven-dried. Detection of reduced carbon products was performed using ^1^H NMR spectroscopy with presaturation of the MeCN and H_2_O signals. 0.2 mL of the CPE solution were injected into an NMR tube with a sealed capillary standard of known concentration of dimethylformamide/C_6_D_6_. No formaldehyde, methanol, ethanol, or other C-containing products were detected.

### X-Ray Structure Determination

X-ray diffraction studies for H4 were carried out on a Bruker Photon100 CMOS diffractometer or a Bruker SMART APEXII diffractometer equipped with a CCD detector.^50^ Measurements were carried out at 100 or 90 K using Mo Kα 0.71073 Å radiation for H4. The crystals were mounted on a Kapton Loop with Paratone-N oil. Initial lattice parameters were obtained from a least-squares analysis of more than 100 centered reflections; these parameters were later refined against all data. Data collected were corrected for Lorentz and polarization effects with Saint,^51^ and absorption using Blessing’s method and merged as incorporated with the program Sadabs.^52^ Space group assignments were based upon systematic absences, E statistics, and successful refinement of the structures. Structures were solved by direct methods with the aid of successive difference Fourier maps and were refined against all data using the SHELXT and SHELXL-2014 software package.^53^ Thermal parameters for all non-hydrogen atoms were refined anisotropically. Hydrogen atoms, where added, were assigned to ideal positions and refined using a riding model with an isotropic thermal parameter 1.2 times that of the attached carbon atom (1.5 times for methyl hydrogens).

### Other Physical Measurements

^1^H NMR, ^13^C NMR and ^31^P NMR spectra were recorded at ambient temperature using a Varian 600 MHz spectrometer, a Bruker 400 MHz TopSpin spectrometer, or a Bruker 800 MHz TopSpin spectrometer equipped with a cryoprobe, and chemical shifts were referenced to the residual solvent peaks. ^31^P NMR spectra were referenced using an external H_3_PO_4_ standard (chemical shift of H_3_PO_4_ = 0 ppm). Combustion analyses were determined by the Microanalytical Lab at the University of California Berkeley. Quantitative measurement of H_2_ was performed on a Varian 3800 GC equipped with a TCD detector and a Carboxen 1010 PLOT fused silica column (30 m × 0.53 mm, Supelco) using dinitrogen (99.999%, Praxair) as the carrier gas. H_2_ concentration was determined using a previously prepared working curve. IR spectra were recorded in a sealed liquid cell (SPECAC) on a Bruker Alpha IR spectrometer.

## Data Availability

The data underlying this study are available in the published article and its Supporting Information.

## Supporting Information Available

Experimental methods, calculations, CPE results, CV voltammograms, NMR spectra, and pulse radiolysis measurements (PDF)
