# Supplementary material for: Catalyst Protonation Changes the Mechanism of Electrochemical Hydride Transfer to CO2
Source: ACS Org Inorg Au. 2024 Oct 4;4(6):649–57. doi: 10.1021/acsorginorgau.4c00041 (PMC11621949; doi:10.1021/acsorginorgau.4c00041)
Supplement: Supplementary file 1 — gg4c00041_si_001.pdf [file gg4c00041_si_001.pdf]

Supporting Information to Accompany:

Catalyst Protonation Changes the Mechanism of Electrochemical Hydride Transfer to CO_2

Kevin Y. C. Lee, Dmitry E. Polyansky, David C. Grills, James C. Fettinger, Marcos Aceves, Louise A. Berben*

Department of Chemistry, University of California, Davis, CA 95616, USA

Chemistry Division, Brookhaven National Laboratory, Upton, NY 11973-5000, USA

email to: laberben@ucdavis.edu

Table of Contents

1. Calculations

Calculation S1. Determination of hydricity for H**4**
Calculation S2. Pulse Radiolysis data analysis.
Calculation S3. Calculation of moles of H_2
Calculation S4. Calculation of $HCOO^-$ yield
Calculation S5. Determination of reaction order

2. Tables

Table S1. Crystallographic data for H**4**
Table S2. Selected bond lengths and angles for H**4**, Et_4N-**A**, and H**A**.
Table S3. CPE results for clusters **1**⁻, **2**⁻, H**4** under CO_2 in in MeCN/H_2O (95:5)
Table S4. CPE results for clusters **1**⁻, **2**⁻, H**4** under N_2 in MeCN/H_2O (95:5)

3. Figures

Figure S1. IR of H**4** in MeCN and Toluene
Figure S2. 400 MHz ^{1}H NMR spectra of H**4** in $CDCl_3$
Figure S3. 400 MHz ^{1}H NMR spectra of H**4** in C_6D_6
Figure S4. 400 MHz ^{31}P-NMR of H**4** in C_6D_6
Figure S5. 400 MHz ^{13}C-NMR of H**4** in C_6D_6
Figure S6. Solid-state structure of H**4**
Figure S7. IR and Calibration curve used to determine pK_a of H**A**
Figure S8. Data collected for **A**⁻ using TRPR-IR experiments.
Figure S9. Data collected for H**4** using TRPR-IR experiments.
Figure S10. CV's of H**4** under N_2, CO, or excess PEt_3
Figure S11. Calibration curve used to quantify formate
Figure S12. GC-TCD spectrum and calibration curve used to quantify H_2
Figure S13. Charge vs time plot for CPE of H**4** in MeCN/H_2O (95:5), and control experiments.
Figure S14. 600 MHz ^{13}C-NMR detection of $HCOO^-$ produced from a CPE using $^{13}CO_2$.
Figure S15. IR spectra of 0.3 mM H**4** before and after CPE to show stability
Figure S16. Variable scan rate data collected under 1 atm N_2 in MeCN from 0.05 – 5 Vs^{-1}.
Figure S17. Plot of j vs [H_2O] with H**4** used to determine rate of reaction with respect to H_2O under N_2 and CO_2 in MeCN
Figure S18. Plot of j vs [H**4**] used to determine rate of reaction with respect to H**4** under N_2 in MeCN/H_2O (95:5)

4. References

1. CALCULATIONS

Calculation S1. Determination of hydricity for H**4**

$H4 \rightleftharpoons 4^{+} + H^{-}$	$\Delta G^{\circ}_{H-}(H4)$
$AnH^{+} \rightleftharpoons An + H^{+}$	$\Delta G = -RT\ln(K_a)$
$H^{-} + H^{+} \rightleftharpoons H_2$	$-\Delta G^{\circ}_{H-}(H_2)$
$H4 + AnH^{+} \rightleftharpoons 4^{+} + An + H_2$	ΔG_r

$\Delta G_r = \Delta G^{\circ}_{H-}(H4) - RT\ln(K_a) - \Delta G^{\circ}_{H-}(H_2)$

Since no reaction was observed between H4 and AnH^{+},

$\Delta G_r > 0$

Therefore,

$\Delta G^{\circ}_{H-}(H4) - RT\ln(K_a) - \Delta G^{\circ}_{H-}(H_2) > 0$

Thus,

$\Delta G^{\circ}_{H-}(H4) > RT\ln(K_a) + \Delta G^{\circ}_{H-}(H_2)$

Since the pK_a of AnH^{+} is 10.6 in MeCN and $\Delta G^{\circ}_{H-}(H_2)$ is 76.0 kcal/mol in MeCN, at a temperature of 298.15 K,

$\Delta G^{\circ}_{H-}(H4) > 0.5925\ln(10^{-10.6}) + 76.0$

Therefore,

$\Delta G^{\circ}_{H-}(H4) > 61.54$ kcal/mol

Calculation S2. Pulse Radiolysis data analysis.

An electron transfer equilibrium between a reference compound (Ref) with a known reduction potential (E_{Ref}) and a cluster (Fe4) with unknown potential (E_{Fe4}) is shown in eqn. S4:

$$Ref^{\bullet-} + Fe4 \rightleftarrows Ref + Fe4^{\bullet-} \quad (S4)$$

Under pulse radiolysis conditions where only a small fraction (ca. 1 – 2 μM) of added starting materials with initial concentrations $[Ref]_0$ and $[Fe4]_0$ are converted to radical anions, the equilibrium constant can be described by eqn. S5:

$$K_{eq} \approx \frac{[Ref]_0}{[Fe4]_0} \times \frac{Abs\ (Ref^{\bullet -}) - Abs(Ref \ldots Fe4^{\bullet -})}{Abs(Ref \ldots Fe4^{\bullet -}) - Abs(Fe4^{\bullet -})} \approx \frac{[Ref]_0}{[Fe4]_0} \times R \qquad \text{(S5)}$$

where the ratio R is defined as:

$$R = \frac{Abs\ (Ref^{\bullet -}) - Abs(Ref \ldots Fe4^{\bullet -})}{Abs(Ref \ldots Fe4^{\bullet -}) - Abs(Fe4^{\bullet -})} \qquad \text{(S6)}$$

Abs($Ref^{\bullet-}$) is the absorbance of the Ref radical anion measured in the absence of Fe4; Abs($Fe4^{\bullet-}$) is the absorbance of the Fe4 radical anion measured in the absence of Ref, and Abs(Ref…$Fe4^{\bullet-}$) is the absorbance of both Ref and Fe4 radical anions measured in the presence of known concentrations of $[Ref]_0$ and $[Fe4]_0$. For a derivation of eqns. S4 and S5 see previous publication.[1]

The reduction potential of Fe4 can be obtained from K_{eq} as follows:

$$E_{Fe4} = E_{Ref} - 0.059 \times \log(K_{eq}) = E_{Ref} - \Delta E \qquad \text{(S7)}$$

	Reference	K_{eq}	ΔE, mV	E, V vs $Fc^{+/0}$	E, V vs SCE
**H4** 1 µs	DCB [a]	0.6 ± 0.1[b]	− 13 ± 3	− 2.107 ± 0.003	− 1.707 ± 0.003
**H4** 20 µs	DCB [a]	0.2 ± 0.1[b]	− 39 ± 2	− 2.081 ± 0.002	− 1.681 ± 0.002
**A** 80 µs	$[Ru(bpy)_3](PF_6)_2$	0.04 ± 0.003[b]	− 82 ± 4	− 1.638 ± 0.004	− 1.238 ± 0.004

[a] The $E_{1/2}$ of 1,4-dicyanobenzene (DCB) was determined as – 2.12 V vs. $Fc^{+/0}$ by cyclic voltammetry. [b] Errors in equilibrium constants are obtained from at least three independent measurements and propagated to errors in reduction potentials according to conventional error propagation rules.

Calculation S3. Calculating moles of H_2 made in CPE experiment.

We used the ideal gas law for this calculation.

$$n = \frac{PV}{RT} = \frac{(Area\ of\ GC\text{−}TCD\ Signal \times \%H_2)(Volume\ CPE\ cell - Volume\ Solvent)}{0.08206 \times 296} \qquad \text{(S8)}$$

n: number of moles of H_2

P: pressure of H_2 gas (atm) calculated from peak area of GC signal due to H_2 gas. Obtained from calibration curve shown in Figure S11.

V: volume of gas (L), the volume of the cell that is not occupied by liquid.

R: gas constant (0.08206 $L atm K^{-1} mol^{-1}$)

T: temperature (296 K)

Calculation S4. Calculating moles of formate made in CPE experiment.

A sample of 10 mM DMF in C_6D_6 in a capillary was used as a standard for calculating the concentration of products made in CPE and for NMR locking. The concentration of formate made in a CPE experiment was calculated based on the ratio of the integrals of formate and DMF by using the calibration curve shown in Figure S10.

Calculation S5. Determination of reaction order

$$j_{cat} = nFA[Cat]\sqrt{Dk_{obs}[S]^x} \qquad \text{(S9)}$$

Eqn S9. demonstrates the relationship between current, catalyst, and substrate.[2] Where j_{cat} is catalytic current, n is number of electrons, F is Faraday's constant, A is the electrode surface area (cm^{-2}), [Cat] is catalyst concentration (mol cm^{-3}), D is the diffusion coefficient (cm^2 s^{-1}), k_{obs} is the catalytic rate constant (s^{-1}), [S] is the concentration of general substrate denoted as S (M), and x is the reaction order in S.

2. TABLES

Table S1. Crystallographic data for $[HFe_4N(PEt_3)_4(CO)_8]$ (H**4**).

	H**4**
Formula	$C_{32}H_{61}Fe_4NO_8P_4$
Crystal Size, mm^3	0.232 × 0.176 × 0.145
Formula weight, gmol^{-1}	935.09
Space Group	$P2_1/c$
a, Å	13.1007(17)
b, Å	13.7580(18)
c, Å	23.380(3)
α	90°
β	96.3402(19)°
γ	90°
V, Å^3	4188.3(9)
Z	4
T, K	90(2) K
ρ, calcd, g cm^{-3}	1.483
Refl. collected/2θ_{max}	13027
Unique refl./ $I > 2\sigma(I)$	10562
No. parameters/restrains	458/0
λ, A° /μ (Kα), cm^{-1}	0.71073
R_1/GOF	0.0289/1.029
wR_2 $(I > 2\sigma(I))^a$	0.0621
Residual density, e Å^{-3}	0.842, -0.339

^{a} $R_1 = \sum||F_o|- F_c||/\sum|F_o|$, $wR_2 = \{\sum [w(F_o^2-F_c^2)^2]/ \sum [w(F_o^2)^2]\}^{1/2}$.

Table S2. Selected bond lengths and angles for [$HFe_4N(PEt_3)_4(CO)_8$] (H**4**), $Et_4N[Fe_4N(CO)_{12}]$ (Et_4N-**A**),[3] and [$HFe_4N(CO)_{12}$] (H**A**).[4] See Chart 1 below for atom labelling scheme.

	H**4**	**A**⁻	H**A** [a]
Fe_1-N	1.797(1)	1.775(3)	1.77(1)
Fe_2-N	1.925(1)	1.909(4)	1.92(2)
Fe_3-N	1.920(1)	1.896(4)	1.92(2)
Fe_4-N	1.795(1)	1.768(3)	1.77(1)
Fe_1- Fe_2	2.631(6)	2.5916(7)	2.62(1)
Fe_1- Fe_3	2.619(5)	2.6147(7)	2.62(1)
Fe_2- Fe_3	2.601(5)	2.5064(8)	2.54(2)
Fe_2- Fe_4	2.621(6)	2.6005(6)	2.62(1)
Fe_3- Fe_4	2.622(5)	2.6185(7)	2.62(1)
Fe_2- Fe_1-Fe_3	59.41(1)	57.56(2)	-
Fe_1- Fe_2-Fe_4	86.33(1)	86.03(2)	101 (1)
Fe_1- Fe_3-Fe_4	86.57(1)	85.19(2)	101(1)
Fe_1- N_1-Fe_4	178.97(8)	177.8(2)	178.4(6)
Fe_2- H_1-Fe_3	104.65(1)		
Fe_2-H_1	1.648(2)		
Fe_3 - H_1	1.638(2)		

[a] Hydride not resolved on H**A**[4]

Chart 1. Numbering scheme used in Table S2

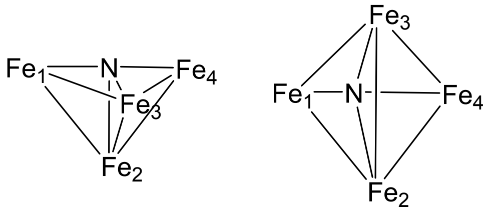

Table S3. CPE results for clusters **1**⁻, **2**⁻ , and H**4**⁻ under CO_2 in 0.1 M Bu_4NBF_4 MeCN/ H_2O (95:5) with 0.3 mM catalyst over 20 minutes. GC working electrode and Pt coil counter electrode.

	Applied *E* /V vs SCE	*q* /C	FE /% HCO_2^-	FE /% H_2
0.3 mM H**4**	−1.52	2.0(0.3)	46(5)	40(5)
blank	−1.52	0.9	nd	14
Rinse Test	−1.52	1.0	nd	12
0.3 mM **1**⁻	−1.48	1.6(0.2)	50(3)	40(5)
blank	−1.48	1.1	nd	12
Rinse Test [a]	−1.48	1.3	nd	20
0.3 mM **2**⁻	−1.74	3.3(0.3)	28(5)	65(8)
blank	−1.74	1.8	nd	16
Rinse Test	−1.74	2.1	nd	17

[a] Rinse Test represents trials when the electrode has been gently rinsed with MeCN and used in a subsequent CPE experiment that did not contain any catalyst in order to check for deposited, catalytically active material.
nd = not detected, detection limit is 0.01 mM for formate.

Table S4. CPE results for clusters **1**⁻, **2**⁻ , H**4** under N_2 in 0.1 M Bu_4NBF_4 MeCN/ H_2O (95:5) with 0.3 mM catalyst over 20 minutes. GC working electrode and Pt coil counter electrode.

	E /V vs SCE	*q* /C	FE /% H_2
0.3 mM H**4**	−1.52	2.2(0.3)	90(5)
blank	−1.52	0.8	14
Rinse Test	−1.52	1.0	12
0.3 mM **1**⁻	−1.48	1.8(0.2)	88(5)
blank	−1.48	1.1	12
Rinse Test [a]	−1.48	1.3	20
0.3 mM **2**⁻	−1.74	3.3 (0.5)	87(5)
blank	−1.74	1.5	15
Rinse Test	−1.74	1.7	18

[a] Rinse Test represents trials when the electrode has been gently rinsed with MeCN and used in a subsequent CPE experiment that did not contain any catalyst in order to check for deposited, catalytically active material. nd = not detected, detection limit is 0.01 mM for formate.

3. FIGURES

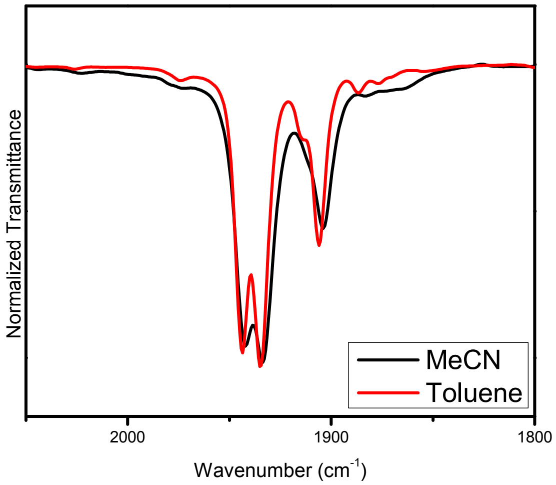

Figure S1. IR spectrum of H**4** in MeCN (red) and toluene (black).

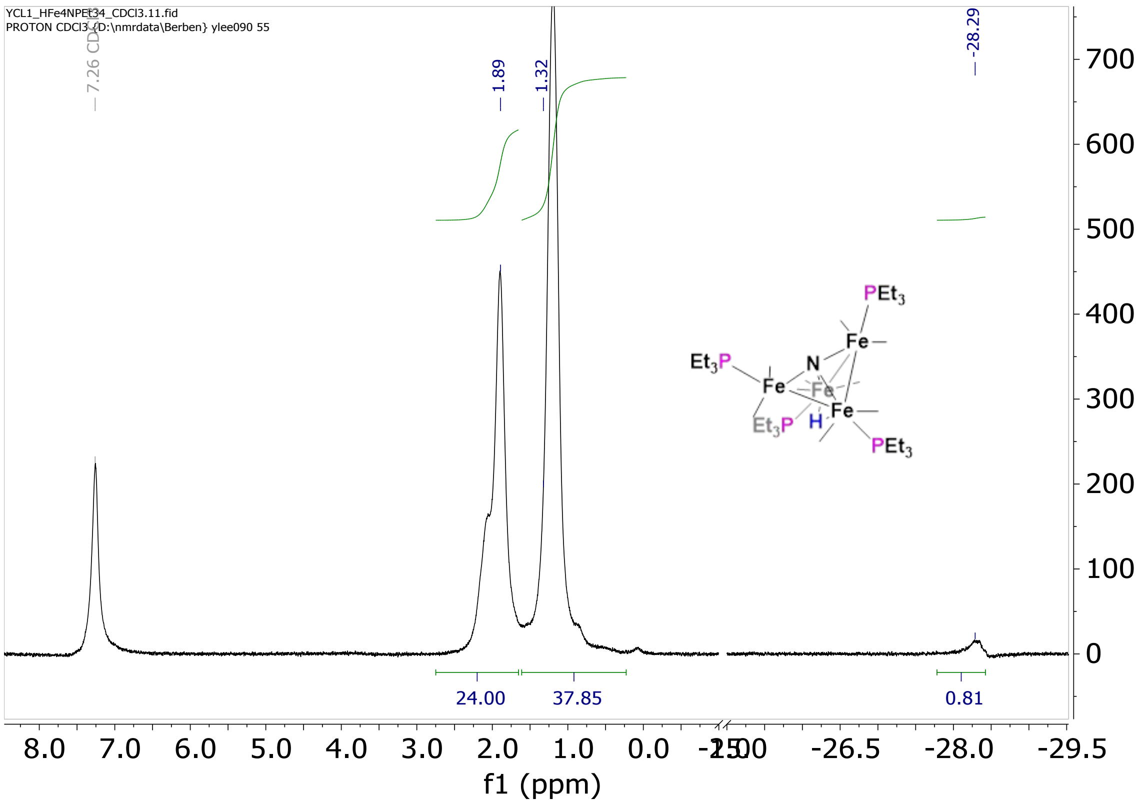

Figure S2. 400 MHz ^{1}H NMR spectrum of (H**4**) in $CDCl_3$.

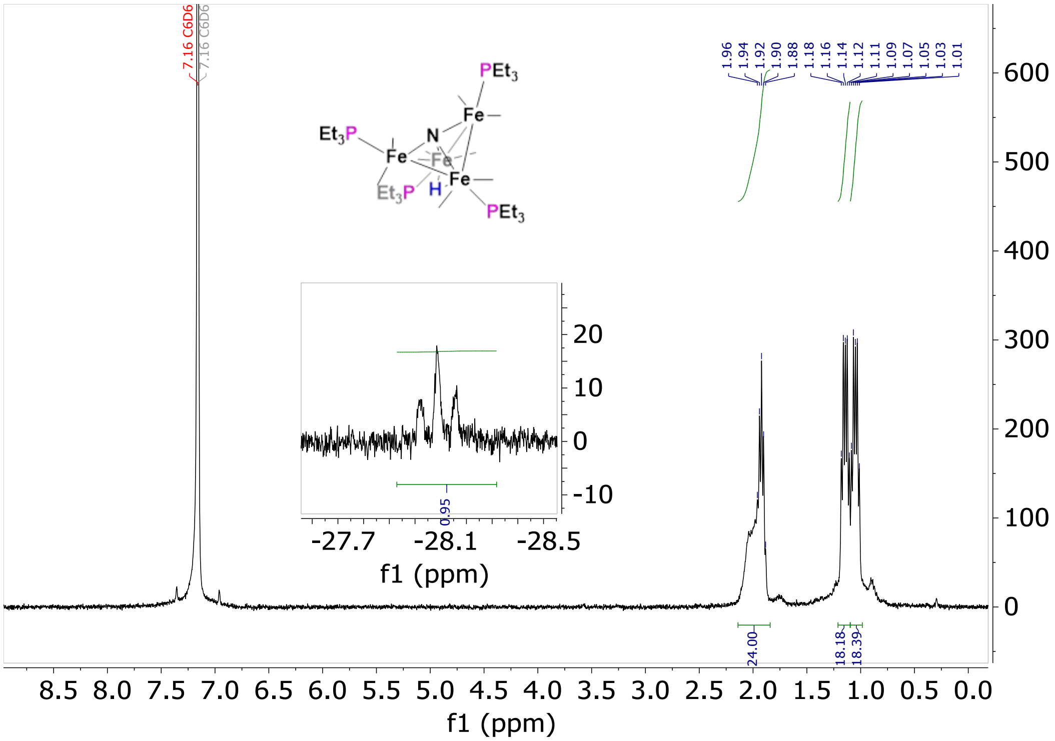

Figure S3. 400 MHz ^{1}H-NMR spectrum of (H**4**) in C_6D_6.

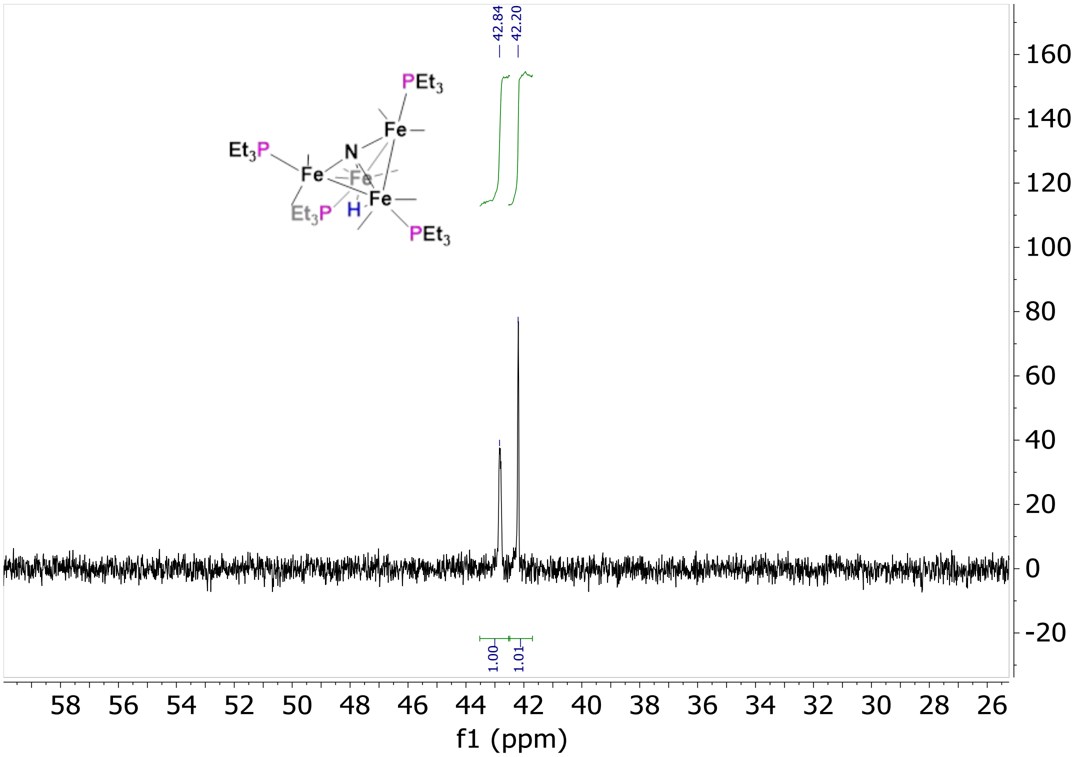

Figure S4. 400 MHz $^{31}P\{^1H\}$-NMR spectrum of (H**4**) in C_6D_6.

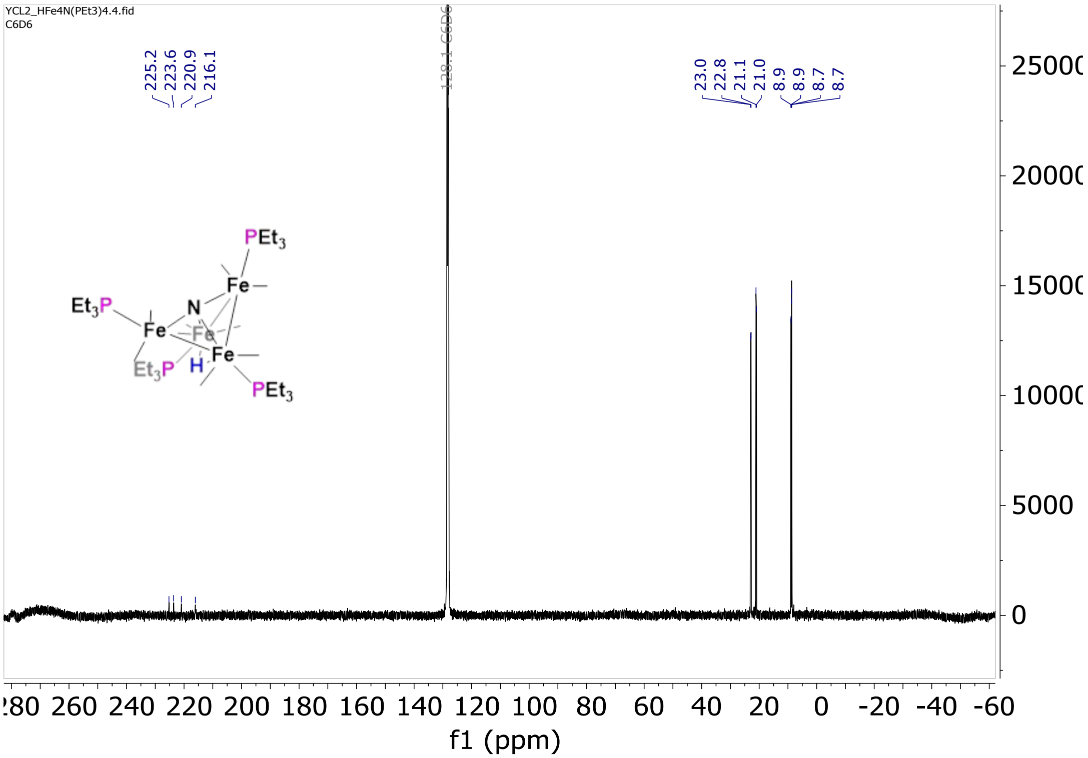

Figure S5. 600 MHz $^{13}C\{^1H\}$-NMR spectrum of (H**4**) in C_6D_6.

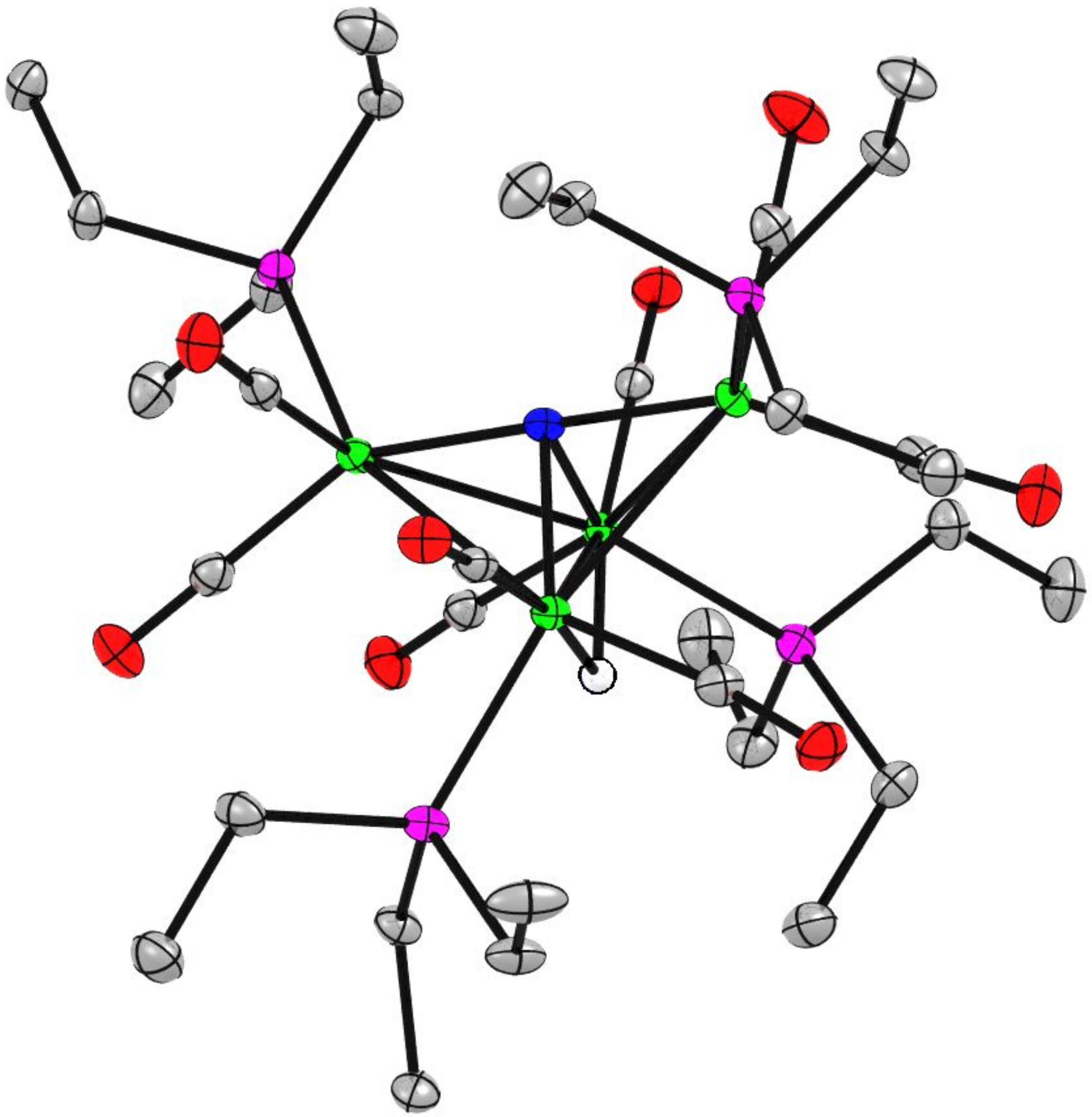

Figure S6. Solid-state structure of H**4**. Green, blue, pink, grey, and red ellipsoids, and white sphere represent Fe, N, P, C, O, and H atoms respectively. Ellipsoids shown at 50%; H atoms omitted except for hydride.

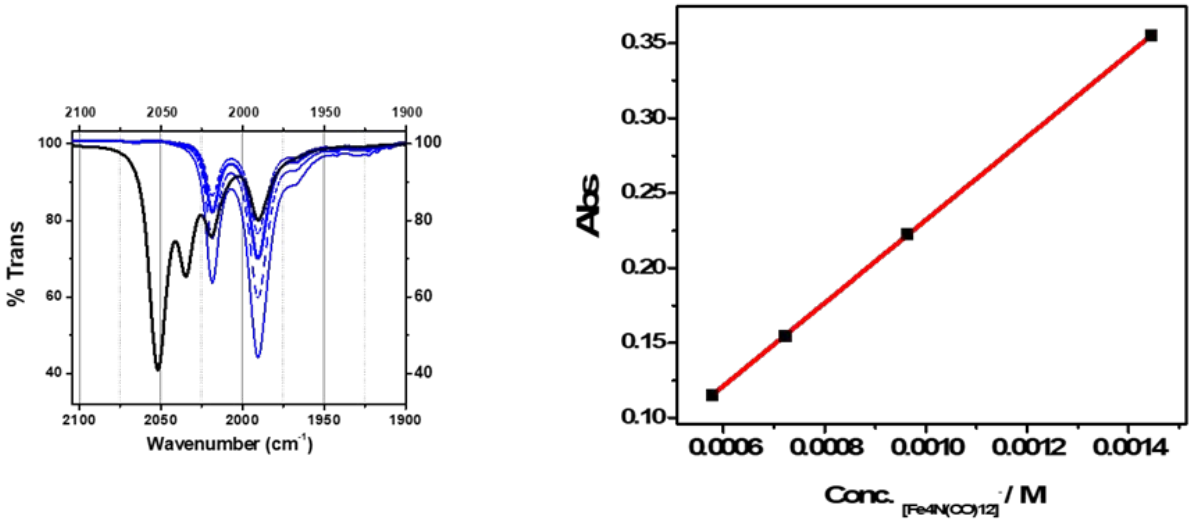

$HFe_4(N)(CO)_{12} \rightleftharpoons [Fe_4(N)(CO)_{12}]^- + H^+$

$pK_a(MeCN) = 3.6$

$$K_a = \frac{[Fe_4N(CO)_{12}{}^-][H^+]}{[HFe_4N(CO)_{12}]}$$

$$pK_a = -\,log_{10}(K_a)$$

Figure S7. IR spectra and calibration curve used to determine pK_a of [$HFe_4N(CO)_{12}$]. (left) IR spectra of [$Fe_4N(CO)_{12}$]$^-$(Blue) and [$HFe_4N(CO)_{12}$] (Black) recorded at different concentrations. This data is used to make the plot at right (right) Plot of Absorbance vs concentration of [$Fe_4N(CO)_{12}$]$^-$, plotted with data read at 1990 cm^{-1} from the left. This calibration curve was used to determine the concentration of [$Fe_4N(CO)_{12}$]$^-$ and [$HFe_4N(CO)_{12}$] that are present in MeCN solution after [$HFe_4N(CO)_{12}$] is dissolved in the solution. (bottom) Determination of the pK_a for [$HFe_4N(CO)_{12}$] using the IR data. The [H^+] was assumed to be equal to the concentration of [$Fe_4N(CO)_{12}$] $^-$, and mass balance was assumed.

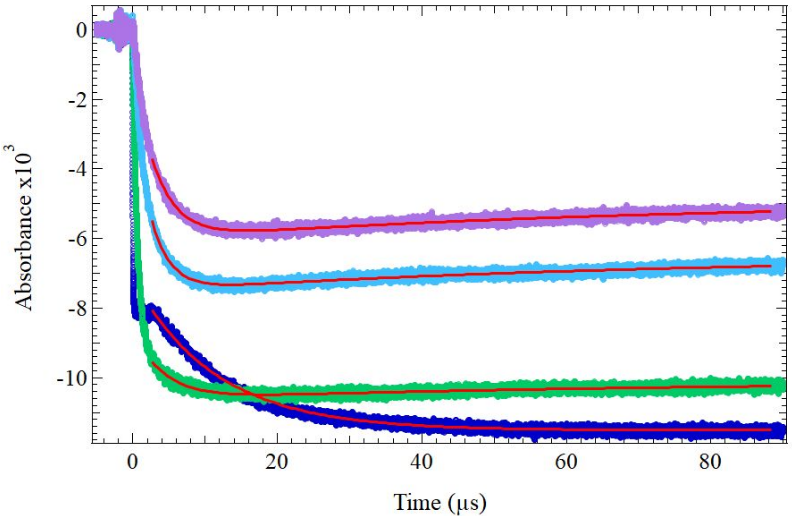

Figure S8A. PR-TRIR transient absorption kinetics recorded at 2014 cm^{-1} after an electron pulse into N_2-saturated CH_3CN solutions containing 50 mM [TBA$^+$ HCO_2^-] and the following: 1.25 mM $Et_4N[Fe_4N(CO)_{12}]$ (blue); 0.375 mM $Et_4N[Fe_4N(CO)_{12}]$ and 1.4 mM $[Ru(bpy)_3](PF_6)_2$ (green); 0.125 mM $Et_4N[Fe_4N(CO)_{12}]$ and 1.8 mM $[Ru(bpy)_3](PF_6)_2$ (light blue); 0.063 mM $Et_4N[Fe_4N(CO)_{12}]$ and 1.9 mM $[Ru(bpy)_3](PF_6)_2$ (purple). Solid red lines represent single exponential fits of kinetic traces.

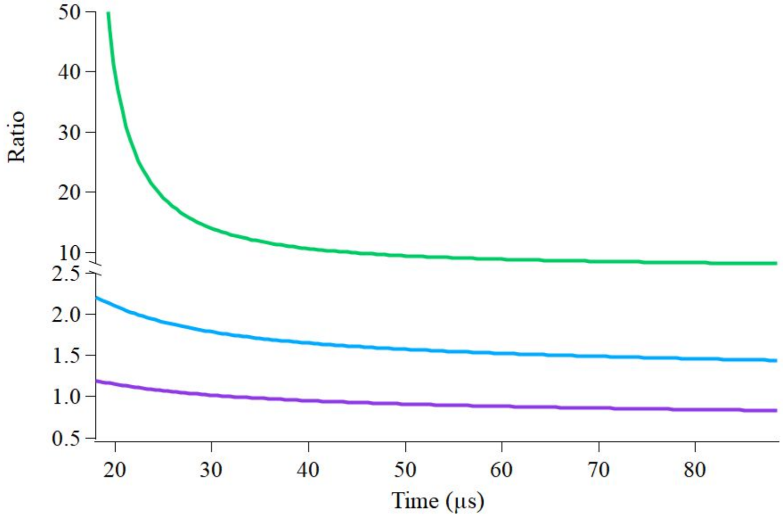

Figure S8B. The ratio of absorbances calculated with eq. S6 using exponential fits of kinetic data shown in Fig. S8A. 0.375 mM $Et_4N[Fe_4N(CO)_{12}]$ and 1.4 mM $[Ru(bpy)_3](PF_6)_2$ (green); 0.125 mM $Et_4N[Fe_4N(CO)_{12}]$ and 1.8 mM $[Ru(bpy)_3](PF_6)_2$ (light blue); 0.063 mM $Et_4N[Fe_4N(CO)_{12}]$ and 1.9 mM $[Ru(bpy)_3](PF_6)_2$ (purple).

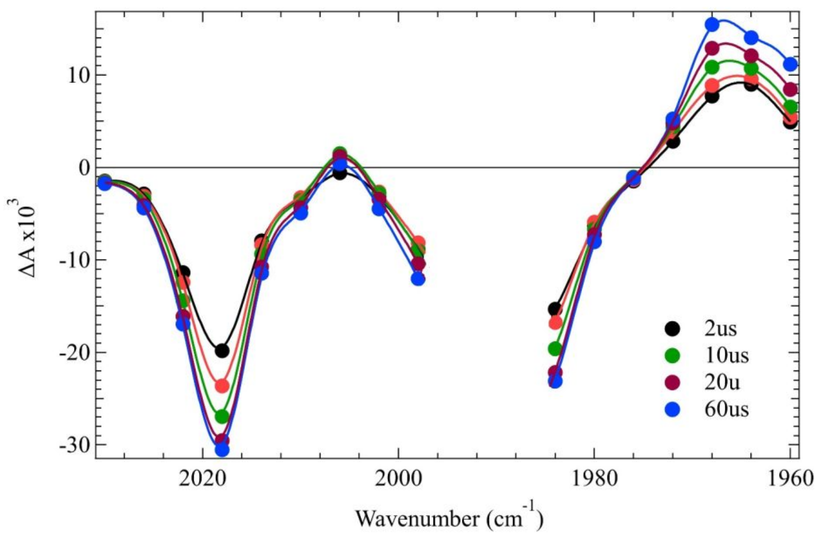

Figure S8C. TRIR spectra recorded between 2 μs and 60 μs after an electron pulse into a N_2-saturated CH_3CN solution containing 1 mM of $Et_4N[Fe_4N(CO)_{12}]$ and 50 mM $[TBA^+ HCO_2^-]$. The gap in the 1985 – 1998 cm^{-1} region is due to the limited output of the probe QCL laser in this region.

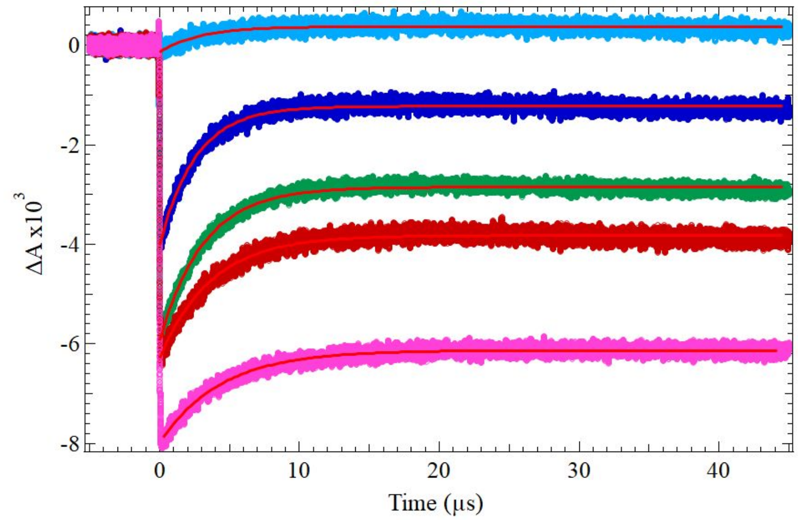

Figure S9A. PR-TRIR transient absorption kinetics recorded at 1935 cm^{-1} after an electron pulse into N_2-saturated CH_3CN solutions containing 50 mM [TBA^+ HCO_2^-] and the following: 1 mM 1,4-dicyanobenzene (light blue); 1 mM [$HFe_4N(PEt_3)_4(CO)_8$] (purple); 0.4 mM 1,4-dicyanobenzene and 0.6 mM [$HFe_4N(PEt_3)_4(CO)_8$] (navy blue); 0.2 mM 1,4-dicyanobenzene and 0.8 mM [$HFe_4N(PEt_3)_4(CO)_8$] (green); 0.1 mM 1,4-dicyanobenzene and 0.9 mM [$HFe_4N(PEt_3)_4(CO)_8$] (dark red). Solid red lines represent single exponential fits of kinetic traces.

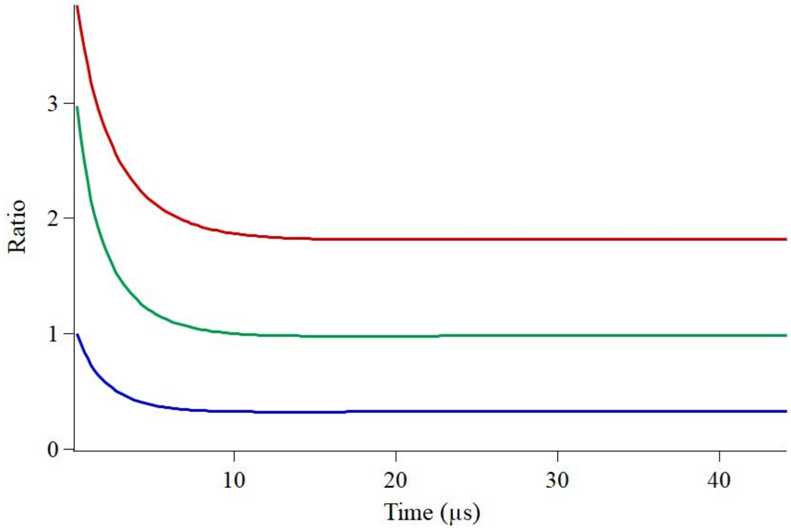

Figure S9B. The ratio of absorbances calculated with eq. S6 using exponential fits of kinetic data shown in Fig. S9A. 0.4 mM 1,4-dicyanobenzene and 0.6 mM [$HFe_4N(PEt_3)_4(CO)_8$] (navy blue); 0.2 mM 1,4-dicyanobenzene and 0.8 mM [$HFe_4N(PEt_3)_4(CO)_8$] (green); 0.1 mM 1,4-dicyanobenzene and 0.9 mM [$HFe_4N(PEt_3)_4(CO)_8$] (dark red).

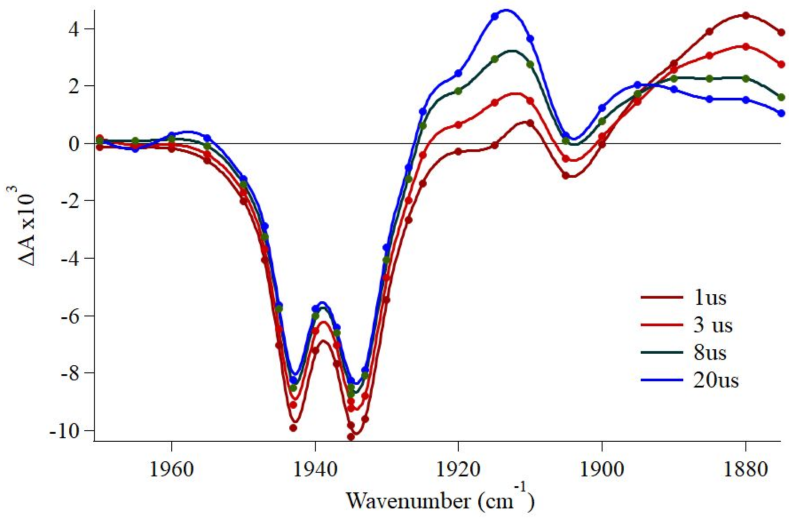

Figure S9C. TRIR spectra recorded between 1 μs and 40 μs after an electron pulse into a N_2-saturated CH_3CN solution containing 1 mM of $[HFe_4N(PEt_3)_4(CO)_8]$ and 50 mM $[TBA^+ HCO_2^-]$.

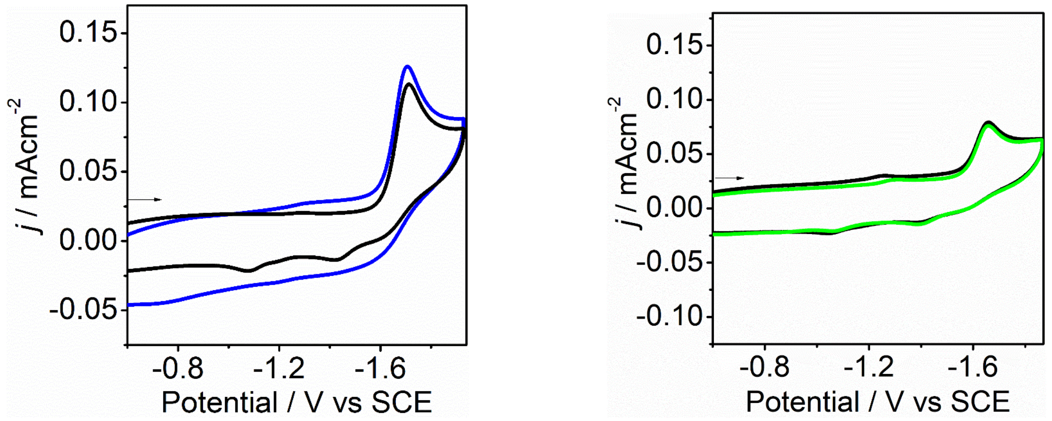

Figure S10. (left) CV's of 0.1 mM H**4** in 0.1 mM $Bu_4N\ BF_4$, MeCN under N_2 (black) and 1 atm CO (blue). (right) CV's of 0.1 mM H**4** in 0.1 mM $Bu_4N\ BF_4$, MeCN solution under 1 atm N_2 (black) and with added 23.4 mM PEt_3 under 1 atm N_2 (green).
All CVs recorded at 0.1 V/s scan rate with a GC working electrode.

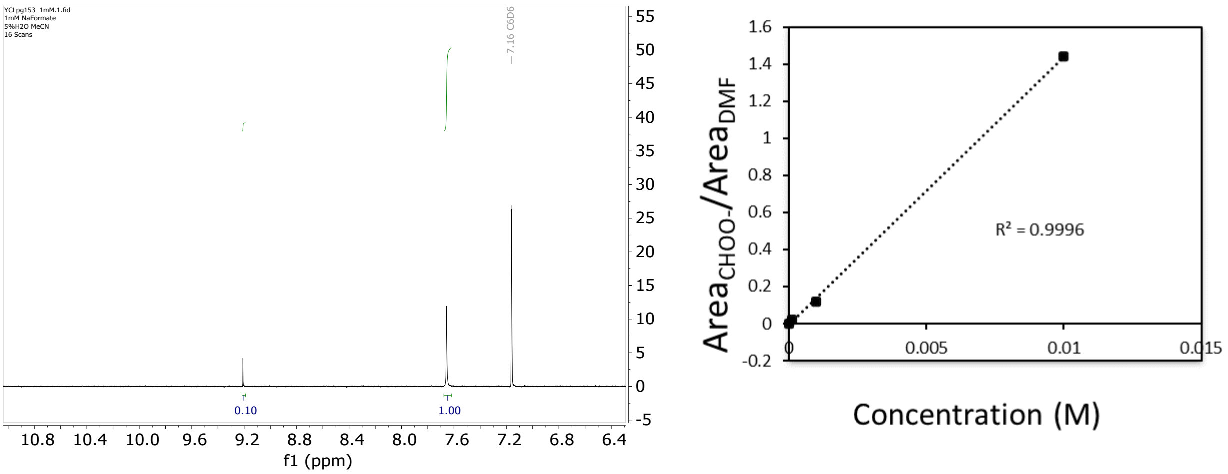

Figure S11. (Left) 600 MHz ^{1}H-NMR spectrum of CPE solution. Formate (9.2 ppm), DMF (7.67 ppm), C_6D_6 (7.16 ppm). (Right) Plot of integration of formate/DMF vs. [sodium formate]. used to quantify formate. Relaxation time was 5 seconds. Calibration curve goes down to 0.001 mM, which is our detection limit for formate.

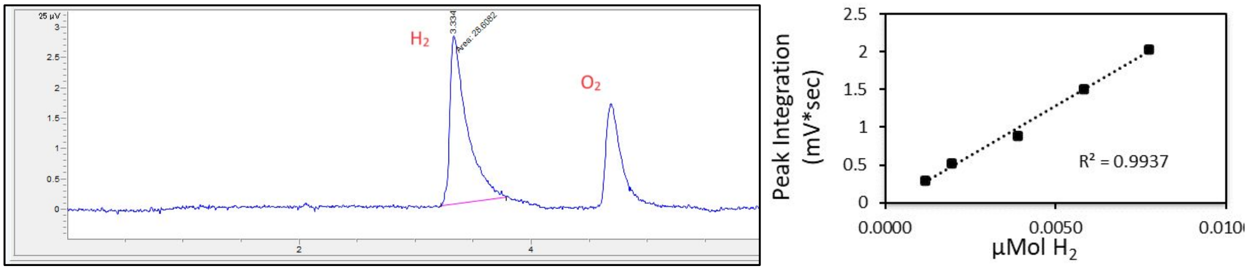

Figure S12. (left) GC-TCD trace of CPE headspace used to quantify H_2. (Right) Calibration curve used to quantify H_2 from GC-TCD data, following CPE.

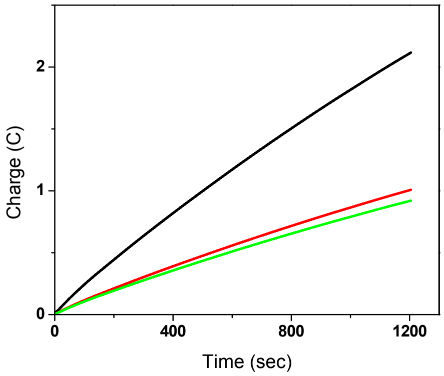

Figure S13. Charge vs time of 0.3 mM H**4**, 0.1 M $Bu_4N\ BF_4$ MeCN under 1 atm CO_2. (Black). Rinse test (red). Blank (Green). Potential is held at the onset of the wave determined by LSV before electrolysis: which is –1.52V vs SCE.

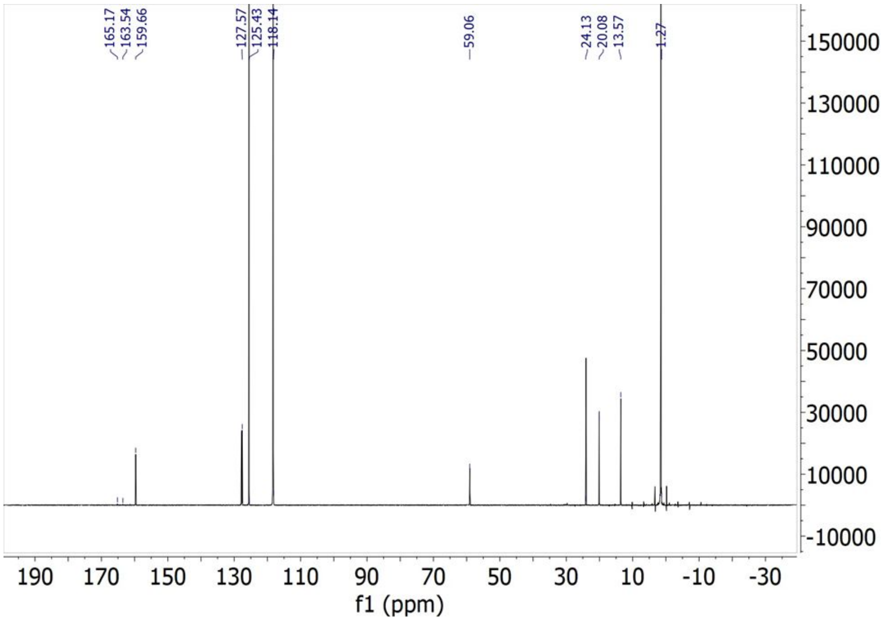

Figure S14. 600 MHz ^{13}C {^1H} NMR spectrum of electrolyte solution after CPE with 0.1 mM H**4** and 5% H_2O in $^{13}CO_2$-saturated solution of MeCN under N_2 with a capillary containing C_6D_6 and DMF as internal standard. Formic acid is observed at 165.2 ppm, C_6D_6 is observed at 127.6 ppm. Bicarbonate $H^{13}CO_3^-$ is observed at 159.7 ppm, $^{13}CO_2$ is observed at 125.4 ppm. 59.1, 24.1, 20.1, 13.6 correspond to the tetrabutylammonium cation. MeCN is observed at 118.1. The peak at 163.5 ppm is DMF.

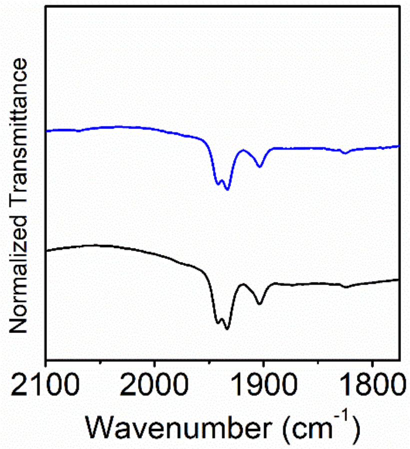

Figure S15. IR spectra of 0.3 mM H**4** in 0.1 M Bu_4NBF_4 MeCN under 1 atm CO_2 of a CPE solution before (black line) and after (blue line) the electrolysis. This shows that H**4** is stable during CPE.

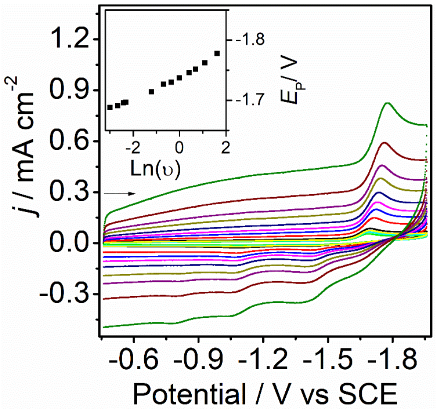

Figure S16. k_{obs} determination of 0.1mM H**4** under 1 atm N_2, 0.1M Bu_4NBF_4 in MeCN. Scan rate was varied from 0.05 V/s to 5 V/s. Insets: Plot of E_p vs Scan Rate (υ).

~~eqn S8 (red line) with −0.5 slope to determine k_{obs}~~. Glassy carbon (GC) working electrode.

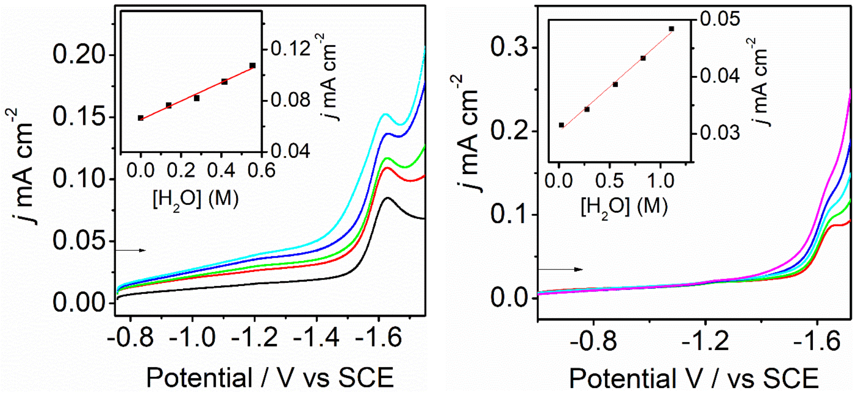

Figure S17. LSV of 0.1 mM H**4** and 0.1 M Bu_4NBF_4 in MeCN under 1 atm of N_2 (left) and 1 atm CO_2 (right). Glassy carbon (GC) working electrode. 0.1 V/s scan rate. Concentration of H_2O was increased to obtain order of reaction. The linear relationship shown in inset plot indicates a second order reaction with respect to acid under N_2 and CO_2. $R^2 = 0.98$ (left), $R^2 = 0.98$ (right).

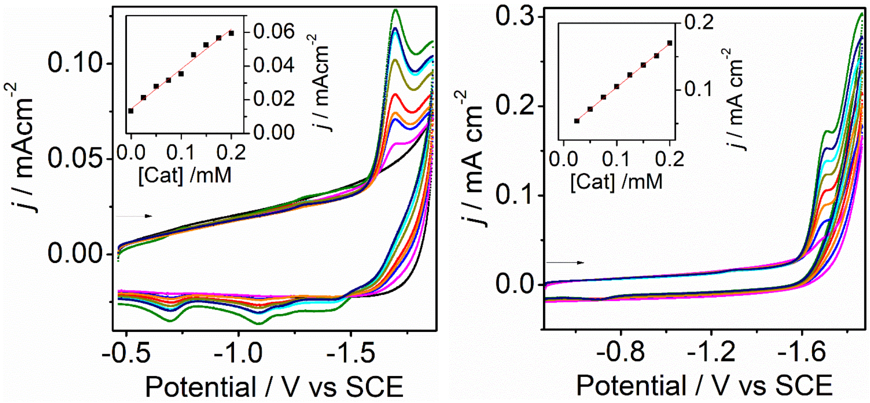

Figure S18. CVs of H**4** and 0.1 M Bu_4NBF_4 in 1:99 H_2O/MeCN under 1 atm N_2 (left), and 1 atm CO_2 (right), used to determine rate of reaction with respect to H**4**. Glassy carbon (GC) working electrode. 0.1 V/s scan rate. Inset is (*j*) vs [H**4**], $R^2 = 0.99$ (left) and $R^2 = 0.99$ (right). The linear relationship shown in inset plot indicates a first-order reaction with respect to [H**4**] in accordance with calculation S5

5. References

[1] Polyansky, D. E.; Manbeck, G. F.; Ertem, M. Z. Combined effects of hemicolligation and ion pairing on reduction potentials of biphenyl radical cations. *J. Phys. Chem. A.* **2023**, 127, 7918–7927. https://doi.org/10.1021/acs.jpca.3c03817.

[2] Savéant, J.; Costentin, C. Elements of molecular and biomolecular electrochemistry; **2019**. https://doi.org/10.1002/9781119292364.

[3] Rail, M. D.; Berben, L. A. Directing the Reactivity of [$HFe_4N(CO)_{12}$]− toward H+ or CO_2 Reduction by Understanding the Electrocatalytic Mechanism. *J. Am. Chem. Soc.* **2011**, 133, 18577–18579. https://doi.org/10.1021/ja208312t.

[4] Tachikawa, M., Stein, J. A., Muetterties, E. L., Teller, R. G., Beno, M. A., Gebert, E., & Williams, J. M. Metal clusters with exposed and low-coordinate nitride nitrogen atoms. *J. Am. Chem. Soc.* **1980** *102*, 6648–6649. https://doi.org/10.1021/ja00541a084
